# Decomposing socioeconomic inequalities in COVID-19 vaccination uptake among Nigerian women of reproductive age: A further analysis of population-based data

**DOI:** 10.1371/journal.pone.0357171

**Published:** 2026-08-28

**Authors:** Michael Ekholuenetale

**Affiliations:** Faculty of Science and Health, School of Health and Care Professions, University of Portsmouth, Hampshire, United Kingdom; Federal University Otuoke, NIGERIA

## Abstract

**Background:**

Understanding COVID-19 vaccine uptake is particularly important in studies of inequity in vaccination access, as differences in uptake may reflect underlying social and structural inequalities. Evidence suggests that socioeconomic status is a key driver for COVID-19 vaccination uptake in Nigeria. This study examines the socioeconomic inequality in COVID-19 vaccination uptake among reproductive-age Nigerian women.

**Methods:**

Data from the 2024 Nigeria Demographic and Health Survey (NDHS) was analyzed for a sample of 36,555 women of reproductive age (15–49 years). COVID-19 vaccination uptake was the outcome variable measured in this study. A socioeconomic decomposition analysis was conducted to identify the factors contributing to inequalities in COVID-19 vaccination uptake across the socioeconomic gradient.

**Results:**

The least disadvantaged women had the highest COVID-19 vaccination uptake (31.6%), whereas the most disadvantaged women had the lowest COVID-19 vaccination uptake (19.2%). The concentration index curve showed that COVID-19 vaccination uptake among Nigerian women was significantly higher among the least disadvantaged group. In addition, having higher education, secondary education, being exposed to mass media, employed, covered by health insurance, being aged 30 years or older, from South West, South South and South East geopolitical zones respectively, were positive contributors to socioeconomic inequality in COVID-19 vaccination uptake.

**Conclusion:**

There was low uptake rate of COVID-19 vaccination among Nigerian women, with approximately one-third of the least disadvantaged, one-quarter of the moderately disadvantaged, and one-fifth of the most disadvantaged women reported receiving a COVID-19 vaccine. This shows that the dream of achieving equitable vaccination coverage will require concerted efforts and targeted strategies that prioritize disadvantaged women, including context-specific policies aimed at reducing socioeconomic barriers to access.

## Introduction

The development of effective and safe vaccines against SARS-CoV-2 has greatly mitigated morbidity and mortality of the COVID-19 pandemic. The coronavirus disease 2019 (COVID-19) pandemic has posed unprecedented challenges to global health systems [1], with vaccination emerging as the most effective strategy for reducing infection, severe illness, and mortality [2,3]. Evidence from previous studies suggests that socioeconomic status is a key driver for COVID-19 vaccination uptake in Nigeria in particular and Sub-Saharan Africa at large [4–6]. Since the introduction of COVID-19 vaccines in 2020, several countries have made significant progress in scaling up the vaccination programme [7]. However, inequitable access and uptake remain major barriers, particularly in low- and middle-income countries (LMICs) [8]. Despite the global efforts to ensure equitable vaccine distribution through initiatives like COVAX [9], disparities in vaccination coverage persist across and within countries, often reflecting underlying socioeconomic inequalities [10].

Nigeria, the most populous country in Africa [11], presents a particularly important context for examining these inequalities. The country has faced longstanding challenges related to health system capacity, uneven distribution of healthcare resources, and high levels of poverty and income inequality. Health financing in Nigeria is largely driven by out-of-pocket expenditure, which disproportionately affects low-income households and limits access to essential health services. These structural inequities have been further exacerbated by the COVID-19 pandemic, which disrupted health services and deepened existing socioeconomic vulnerabilities. Women represent a key population group in the context of COVID-19 vaccination [12,13], particularly in Nigeria where gender norms and socioeconomic conditions significantly influence health-seeking behaviour. Women often face unique barriers to accessing healthcare services, including limited financial autonomy, lower educational attainment, and little decision-making power within households.

COVID-19 vaccination rollout in Nigeria began in early 2021 [14,15], yet coverage has remained relatively low compared to the global targets. Preliminary analyses indicated that only a small proportion of the population had been fully vaccinated during the initial phases of the rollout, highlighting gaps in access and uptake. Studies from West Africa demonstrate that gender can shape COVID-19 vaccination through distinct social and structural pathways. In Ghana, a qualitative study among women in Greater Accra and Ashanti identified fear of adverse effects, vaccine misconceptions, long queues and vaccine shortages as important barriers, while access to information and protecting family members facilitated uptake [16]. In Nigeria, there is evidence that female sex is associated with greater COVID-19 vaccine hesitancy, alongside socioeconomic disadvantage and rural residence [17]. Albeit, a multi-country study found no significant sex difference in self-reported uptake across Nigeria and Senegal, highlighting context-specific gender effects and the need for sex-disaggregated analysis [18].

The COVID-19 pandemic has further amplified gendered health inequalities [12,13]. Nigerian women, especially those in vulnerable situations, experienced heightened socioeconomic and health inequities. These inequities may translate into disparities in access to COVID-19 vaccines, as women in lower socioeconomic strata are more likely to face financial, informational, and logistical barriers. Socioeconomic inequalities in health care are well documented in Nigeria. For example, a study on high-risk fertility behaviour among Nigerian women have shown that the socioeconomic disadvantaged is strongly associated with adverse health outcomes [19], underscoring the importance of addressing inequality in health interventions. These patterns are likely to extend to COVID-19 vaccination uptake, where similar structural determinants influence access and utilization.

Understanding socioeconomic inequality in COVID-19 vaccination uptake among Nigerian women is therefore crucial for several reasons. First, it provides insights into the distributional equity of vaccination programmes and identifies groups that are being left behind. Second, it informs targeted interventions aimed at improving vaccine coverage among disadvantaged populations. Also, it contributes to broader efforts to achieve health equity and universal health coverage, which are central to global health agendas such as the Sustainable Development Goals (SDGs) [20]. Moreover, examining inequality using robust quantitative measures, such as concentration indices and decomposition analysis, can help to disentangle the relative contributions of different socioeconomic factors in observed disparities. Such analyses are essential for designing evidence-based policies that address both demand-side and supply-side barriers to vaccination. It is in the light of the above that this study was conducted to examine the socioeconomic inequalities in COVID-19 vaccination uptake among Nigerian women of reproductive age.

## Methods

### Data source

Of the total 39,050 women of reproductive age recruited during the 2024 Nigeria Demographic and Health Survey (NDHS), 36,555 women of reproductive age (15–49 years) were interviewed and responded to the question on whether or not they have received COVID-19 vaccine. This accounted for 93.6% response rate and analyzed for this study. The National Population Commission (NPC), acted on behalf of the Federal Ministry of Health and Social Welfare (FMoHSW) to support the data collection process, which took place between December 1, 2023, and May 7, 2024. The survey was funded by the United States Agency for International Development’s (USAID). In addition, ICF, the World Health Organisation (WHO), the United Nations Population Fund (UNFPA), the United Nations Children’s Fund (UNICEF), and the Global Fund to Fight AIDS, Tuberculosis, and Malaria (Global Fund) were among the other organisations and agencies that provided financial or technical assistance to enable the survey’s successful implementation. The data analyzed for this study can be accessed here [21].

### Exclusion criteria

Institutional populations, including those in hotels, barracks and prisons, were not included in the survey.

### Sample design

The 2024 NDHS used a two-stage stratified sample design. Nigeria is separated into six administrative zones: North Central, North East, North West, South East, South South, and South West. The Federal Capital Territory (FCT) and 36 states make up the zones, which result in 37 subnational entities. Local government areas (LGAs) make up each state, while communities make up each LGA. During the delineation process for the impending census, each locality is further divided into suitable areas known as enumeration areas (EAs). These EAs served as the basis for primary sampling units (PSUs), also known as clusters in the 2024 NDHS. By dividing each of the 36 states and FCT into urban and rural areas, stratification was accomplished. There were 74 sampling strata in all. In each stratum, samples were chosen separately using a two-stage selection process. The initial step was to choose sample locations, or clusters, made up of EAs. Within each sampling stratum, EAs were selected with a probability that was proportionate to their size. There were 701 urban and 699 rural clusters out of the 1,400 total that were chosen. A systematic sample of households was used in the second step. All of the chosen clusters underwent a household listing procedure, and a systematic equal probability selection process was used to choose a set number of 30 households per cluster, for a total sample size of about 42,000 households. At the time of listing and during interviews, Global Positioning System (GPS) data were gathered for every household. The report of the sampling design, training of field assistants and data collection process has been published previously by NDHS [https://www.dhsprogram.com/data/dataset/Nigeria_Standard-DHS_2024.cfm?flag=1] [22].

### Selection and measurements of variables

#### Outcome variable.

The outcome variable examined in this study was COVID-19 vaccine uptake among Nigerian women of reproductive age. This variable was obtained from the question: “*S1112P: Received COVID-19 vaccination*”. COVID-19 vaccination data was self-reported by the women based on their uptake status during and after the global COVID-19 pandemic. The self-reported uptake was verified with information provided by the respondents on the number of doses received, only those who reported uptake and receipt of ≥1 dose were coded “1” and “0” if otherwise.

#### Creating socioeconomic disadvantage variable.

A socioeconomic disadvantage index was constructed using selected indicators reflecting participants’ social and economic circumstances. The indicators included rural residence, belonging to the poorest household wealth quintile, having no formal education, and not being currently employed. Principal component analysis (PCA) was used to combine these correlated indicators into a single composite measure of socioeconomic disadvantage. Prior to PCA, the component variables were appropriately coded and standardised to ensure comparability across indicators measured on different scales. The first principal component, which captured the greatest proportion of the common variance among the selected indicators, was used to generate an overall socioeconomic disadvantage score for each participant. The resulting scores were standardised as z-scores, with higher values indicating greater socioeconomic disadvantage. Based on the distribution of the standardised scores, participants were subsequently categorised into three groups, as low, medium, and high socioeconomic disadvantage to facilitate analysis of inequalities in COVID-19 vaccination uptake among women of reproductive age in Nigeria. Therefore, higher score indicates being more disadvantaged, while the lower score indicates the less disadvantaged respectively.

#### Explanatory variables.

Age (in years): 15–19, 20–24, 25–29, 30–34, 35–39, 40–44, 45–49; education: none, primary, secondary, higher; religion: Christianity, Islam, traditional/no religion; covered by health insurance scheme: yes vs no; marital status: single, currently married, formerly married; employment status: not working vs working; exposure to media (read newspaper, listen to radio, watch tv, use internet): yes vs no.

The cultural norms about wife-beating was created by aggregating responses from women in each community. The items used include: “beating justified if wife goes out without telling husband”, “beating justified if wife neglects the children”, “beating justified if wife argues with husband”, “beating justified if wife refuses to have sex with husband” and “beating justified if wife burns the food”. A binary variable was created by aggregating responses from women for acceptance of wife beating, where “no” indicates empowered women and “yes” to wife beating justified, indicates women not empowered. The “wife-beating justified” variable was included as a proxy measure of gender-related social norms and women’s empowerment, which may shape access to and uptake of health services. Attitudes that legitimise wife-beating may reflect the internalisation of patriarchal norms and reduced autonomy in household and personal health-related decision-making. These factors are relevant to vaccination because women’s autonomy, access to information, and ability to make independent healthcare decisions can influence vaccine confidence and service utilisation. The World Health Organization identifies limited autonomy and decision-making power as important gender-related barriers to COVID-19 vaccination [23]. Evidence from Nigeria further shows that gender attitudes, including attitudes towards wife-beating, are associated with vaccination uptake, even after accounting for socioeconomic factors [24]. Moreover, Nigerian research has demonstrated that acceptance of wife-beating is socially patterned and associated with broader socioeconomic and demographic characteristics [25]. Accordingly, this variable was included to assess whether gender norms contribute to socioeconomic inequalities in COVID-19 vaccination uptake.

Higher acceptance of wife-beating is considered an indicator of lower women’s empowerment because it reflects the adherence to gender norms that allows male dominance and violence against women. Women who justify wife-beating are generally less likely to perceive themselves as entitled to autonomy, equal decision-making, and freedom from violence, all of which are fundamental dimensions of empowerment. Consequently, greater acceptance of wife-beating is interpreted as lower empowerment, whereas rejection of all justifications for wife-beating is regarded as an indicator of higher empowerment.

Sex of household head: male vs female; household size: 1–2, 3–4, 5–6, 7 + ; household wealth: wealth indicator weights were assigned using PCA. The wealth indicator variables (bicycle, motorcycle/scooter, car/truck, main floor material, main wall material, main roof material, sanitation facilities, water source, radio, television, electricity, refrigerator, cooking fuel, furniture and number of people per room) were standardised and scores were assigned. Factor loadings, or factor coefficient scores, and z-scores were computed. To determine each household’s wealth index value, the indicator values were multiplied by the loadings and added together. The overall scores were divided into the poorest/poorer/middle/richer/richest groups using the standardised z-score.

Since the DHS did not gather aggregate-level data at the community-level, we used EAs to represent communities. Therefore, the analysis’s community-level factors were centred on women’s characteristics, especially those that affect the coverage of COVID-19 vaccinations. Community-level women’s acceptance of wife beating was created from the individual woman cultural norms about wife-beating justification by aggregating responses from women in each community; hence, community-level acceptance of wife beating was categorized as more acceptance (less empowered) if the proportion of women who reported wife beating justified in a given community was 79–100% and less acceptance (more empowered) if the proportion was 0–78% (median = 79%; IQR = 28%; minimum = 0%; maximum = 100%). Community-level willingness for COVID-19 vaccine uptake was created from question S1112S “*Willing to be vaccinated against COVID-19*” and categorized as high if the proportion of women who reported willingness to COVID-19 vaccine uptake in a given community was 47–100% and low if the proportion was 0–46% (median = 47%; IQR = 34%; minimum = 0%; maximum = 100%). Community-level nativity was categorized as high if the proportion of women who had lived 5 years or more in a given community was 88–100% and low if the proportion was 0–87% (median = 88%; IQR = 17%; minimum = 0%; maximum = 100%). Community-level urbanicity was categorized as high if proportion of women who reside in urban areas was 50–100% and low if the proportion was 0–49% (median = 50%; IQR = 50%; minimum = 0%; maximum = 100%). Community-level illiteracy was categorized as high if proportion of women who cannot read at all in a given community was 40–100% and as low if the proportion was 0–39% (median = 40%; IQR = 59%; minimum = 0%; maximum = 100%). Community-level media use was categorized as high if the proportion of women who are exposed to media use in a given community was 66–100% and as low if the proportion was 0–65% (median = 66%; IQR = 58%; minimum = 0%; maximum = 100%). Community-level poverty was categorized as high if the proportion of women from the two lowest wealth quintiles in a given community was 34–100% and low if the proportion was 0–33% (median = 34%; IQR = 74%; minimum = 0%; maximum = 100%). Geopolitical zone: North West, North East, North Central, South East, South South, South West. This approach was used in a previous study [26–28].

#### Cut-offs and rationale for constructing community-level variables.

The community-level variables were derived from individual-level responses using an aggregation approach consistent with standard analytical procedures in demographic and public health research. DHS used a hierarchical sampling design in which women are nested within households and households are nested within clusters or primary sampling units (PSUs), which are often used as proxies for communities. As women living within the same cluster are likely to share similar social norms, socioeconomic conditions, cultural practices, and access to health information and services, aggregating individual characteristics at the cluster level provides an appropriate measure of contextual or community influences beyond individual-level effects. This use of cluster-level aggregates of individual responses to characterise the social context is an established, if necessarily approximate, strategy in multilevel social-epidemiological research [29–32]. Because such derived measures cannot, by themselves, fully separate contextual from compositional effects, the approach entered each individual-level characteristic into the model simultaneously with its community-level aggregate, which is the recommended approach for distinguishing, as far as cross-sectional data allow, the contextual contribution from the compositional one [33].

Creating the community-level variables involved calculating the proportion of women within each DHS cluster who possessed a specific characteristic or behaviour of interest. For example, community-level women’s acceptance of wife beating was generated by aggregating women’s responses regarding justification of wife beating within each cluster. Similarly, community-level willingness for COVID-19 vaccine uptake, nativity, urbanicity, illiteracy, media use, and poverty were created by computing the cluster-level proportions of women exhibiting those attributes. This aggregation strategy is statistically justified because it transforms individual responses into contextual indicators reflecting the prevailing social environment within a community.

Based on the aggregation, the resulting community-level variables were categorized into “high” and “low” groups using empirically derived cut-offs based on the distribution of cluster-level proportions across the study population. Specifically, the cut-points corresponded to the median of the aggregated variables, a commonly applied strategy in DHS-based multilevel studies when no universally established thresholds exist. These cut-points were defined a priori from the empirical distribution of each variable and were not selected by reference to the outcome; this distinction matters because it is outcome-optimised cut-points, rather than distribution-based ones, that introduce the serious bias described in the methodological literature [34]. Furthermore, inclusion of these contextual variables provides insights into how shared environmental and sociocultural conditions shape women’s health behaviours and outcomes. Overall, the derivation of community-level variables from aggregated DHS cluster responses represents a robust and widely accepted statistical strategy. This approach was used in a previous study [26–28].

### Analytical approach

The survey module’s (‘svy’) function was utilised to account for sampling design (weighting, clustering, and stratification) since the study employed multi-stage stratified cluster sample design data. Multicollinearity was assessed using the variance inflation factor, which is known to seriously impair logit models [35]. There were no missing data for the variables extracted for analysis. Percentage and chi-square test were conducted in the univariate and bivariate analyses. Stata software, version 17.0 (Stata Corporation, College Station, Texas, USA), was used to analyse the data.

The concentration index was used in estimating the socioeconomic inequality in COVID-19 vaccination. This study employed the Erreygers normalised concentration indices to evaluate the extent of socioeconomic inequalities in COVID-19 vaccination uptake [36]. To determine the contributions of women’s health indicator determinants, the Erreygers Normalised Concentration Index was decomposed [37,38]. Each explanatory factor’s contribution to inequalities in COVID-19 vaccination was broken down into its component parts by health elasticity. The models for identification and estimation of measurement of inequity [39], adapted in this study have been provided.

Logistic regression was used to model the binary outcome of COVID-19 vaccination uptake. However, because the decomposition framework based on the Wagstaff concentration index requires a linear additive specification, the decomposition was not performed using the raw logistic regression coefficients (log odds). Instead, average marginal effects (AMEs) derived from the logistic regression model were used as the partial effects in the decomposition analysis [40]. Thus, the coefficients entered into the Erreygers decomposition model represent marginal effects on the probability scale rather than raw log-odds coefficients. For the binary outcome, a logistic regression model was fitted to estimate the probability of COVID-19 vaccination uptake. Average marginal effects were subsequently used as the partial effects in the Wagstaff decomposition of the concentration index.


$$\begin{array}{c} y_{i}=a+\sum_{k}\beta_{k}X_{ki}+\varepsilon_{i} \end{array}$$

Wagstaff *et al*., [38] show the concentration index for any health measure that has a linear relationship with a set of k exploratory variables may be divided as follows:


$$\begin{array}{c} CI=\sum_{k}\left(\frac{\beta_{k}{\dot{\text{x}}}_{k}}{\hat{y}}\right){CI}_{k}+\frac{{GC}_{\varepsilon }}{\hat{y}} \end{array}$$

Where: β_k_ is the partial

ŷ is the mean of the health variable

ẋ_k_ is the mean of x_k_

CI_k_ denotes the concentration index of x_k_ against education

GC_ɛ_ is the generalised concentration index for the error term

To decompose the Erreygers concentration index, we modified this Equation as shown below [39]:

The Erreygers decomposition of the corrected concentration index (ECI) for a bounded health variable. The complete decomposition, including the Erreygers correction factor [36], is:


$$\begin{array}{c} E_{c}={\text{4}}_{\mu }C_{E} \end{array}$$

Where the Erreygers-corrected concentration index is defined as:


$$\begin{array}{c} E_{c}=\frac{{\text{4}}_{\mu }}{b-a}C, \end{array}$$

When the lower bound is a = 0. For a binary outcome (a = 0, b = 1),


$$\begin{array}{c} E_{c}={\text{4}}_{\mu }C \end{array}$$

The decomposition is then;


$$\begin{array}{c} E_{c}=\text{4}\left[\sum_{k=\text{1}}^{k}\left(\beta_{k}{\;\overset{―}{x}}_{k}\right)CI_{K}+GC_{\varepsilon }\right] \end{array}$$

or, expressing the correction factor explicitly for a bounded variable with lower bound a and upper bound b,


$$\begin{array}{c} E_{c}=\frac{{\text{4}}_{\mu }}{b-a}\left[\sum_{k=\text{1}}^{k}\left(\beta_{k}{\;\overset{―}{x}}_{k}\right)CI_{K}+GC_{\varepsilon }\right] \end{array}$$

When decomposition is written in terms of elasticities, the equivalent expression is:


$$\begin{array}{c} E_{c}={\text{4}}_{\mu }\left[\sum_{k=\text{1}}^{k}\left(\frac{\beta_{k}{\;\overset{―}{x}}_{k}}{\mu }\right)CI_{K}+\frac{GC_{\varepsilon }}{\mu }\right] \end{array}$$

Where;

$E_{c}$: Erreygers normalised (corrected) concentration index

μ : Mean of the health outcome

*a*, *b*: Lower and upper bounds of the health variable

$\beta_{k}$: Partial effect (or average marginal effect, AME, if using a nonlinear model) of determinant ${\;x}_{k}$

${\overset{―}{x}}_{k}$: Mean of determinant ${\;x}_{k}$

$CI_{K}$: Concentration index of determinant ${\;x}_{k}$

$GC_{\varepsilon }$: Generalised concentration index for the error term

For a health outcome which is binary and estimated in a logistic model, the decomposition used average marginal effects (AMEs) instead of the raw logistic coefficients. The specification is:


$$\begin{array}{c} E_{c}=\text{4}\left[\sum_{k=\text{1}}^{k}\left({AME}_{k}\;\overset{―}{x}\right)CI_{K}+GC_{\varepsilon }\right], \end{array}$$

Where;


$$\begin{array}{c} {AME}_{k}=\partial E(y/X)/\partial x_{k} \end{array}$$

In this study, average marginal effects (AMEs), not raw logistic coefficients, were entered as βₖ in the decomposition analysis.

### Ethical consideration

The National Health Research Ethics Committee (NHREC) and the ICF Institutional Review Board approved the survey protocol, methodology and instruments. After all questionnaires were finalised in English, they were translated into Hausa, Yoruba, and Igbo. The 2024 NDHS employed computer-assisted personal interviewing to collect data. This study used the de-identified public secondary dataset in accordance with established ethical guidelines; NDHS obtained the respondents’ informed consent; no additional participant agreement or consent was required because the authors were granted permission to use the data. The details of ethical guidelines can be found here: http://goo.gl/ny8T6X.

## Results

Table 1 shows the pattern of COVID-19 vaccination uptake by socioeconomic status. The uptake of COVID-19 vaccination among Nigeria women was generally low. Across the levels of women’s characteristics, COVID-19 vaccination uptake was higher among the least disadvantaged in contrast to the most disadvantaged women. See Table 1 for details.

**Table 1:** Weighted COVID-19 vaccination uptake among least disadvantaged, moderately disadvantaged and most disadvantaged women.

Variable	% of respondents (n = 36,555)	COVID-19 vaccination uptake by socioeconomic gradient		
		Least disadvantaged (n = 12,208 – unweighted)	Moderately disadvantaged (n = 12,182 – unweighted)	Most disadvantaged (n = 12,165 – unweighted)
**Age in 5-year groups**				
15-19	20.2	17.7	19.0	14.5
20-24	17.1	31.2	25.1	17.6
25-29	16.3	30.4	27.3	18.4
30-34	14.4	33.7	31.1	23.2
35-39	13.0	36.1	32.6	21.4
40-44	10.7	39.6	33.4	22.0
45-49	8.3	40.9	36.0	22.6
P		<0.001*	<0.001*	<0.001*
**Highest educational level**				
No education	29.6	27.7	23.4	16.3
Primary	11.2	33.5	25.4	24.7
Secondary	43.8	28.2	26.4	26.6
Higher	15.3	38.4	38.3	35.7
P		<0.001*	<0.001*	<0.001*
**Religion**				
Christianity	50.4	28.3	27.2	24.6
Islam	49.1	41.8	28.6	18.3
Traditiona/no religion	0.5	28.6	12.3	12.6
P		<0.001*	0.005*	<0.001*
**Covered by health insurance**				
No	96.6	30.6	27.1	19.1
Yes	3.4	45.8	45.3	43.1
P		<0.001*	<0.001*	<0.001*
**Marital status**				
Single	29.3	24.8	22.2	17.5
Currently married	65.5	35.6	30.3	19.2
Formerly married	5.2	34.7	34.1	26.3
P		<0.001*	<0.001*	<0.001*
**Employment status**				
Not working	40.4	21.8	22.2	15.0
Working	59.6	35.1	31.0	24.6
P		<0.001*	<0.001*	<0.001*
**Wife beating justified**				
No	78.9	31.8	27.7	19.7
Yes	21.1	29.3	27.8	18.2
P		0.072	0.910	0.044*
**Exposed to media**				
No	34.8	27.5	24.8	16.7
Yes	65.2	31.9	28.7	25.4
P		0.006*	<0.001*	<0.001*
**Sex of household head**				
Male	78.9	31.2	27.8	18.8
Female	21.1	32.4	27.2	22.9
P		0.190	0.520	0.002*
**Household wealth**				
Poorest	17.1	20.9	28.3	15.9
Poorer	16.3	30.4	26.5	19.7
Middle	19.9	31.0	28.0	26.6
Richer	23.1	33.4	27.9	22.1
Richest	23.6	30.6	27.7	26.0
P		0.016*	0.784	<0.001*
**Household size**				
1-2	10.3	33.5	28.6	14.1
3-4	25.3	32.9	28.1	17.8
5-6	27.0	31.0	29.1	19.6
7+	37.3	29.1	26.1	20.1
P		0.002*	0.024*	<0.001*
**Community-level acceptance of wife-beating**				
More acceptance (less empowered)	41.3	29.0	27.6	17.8
Less acceptance (more empowered)	58.7	32.0	27.7	22.5
P		0.012*	0.940	<0.001*
**Community-level willingness for COVID-19 vaccine uptake**				
Low	50.4	17.3	16.8	7.4
High	49.6	47.1	37.4	31.8
P		<0.001*	<0.001*	<0.001*
**Community-level nativity**				
Low	37.2	28.6	24.7	21.0
High	62.8	35.5	29.4	18.8
P		<0.001*	<0.001*	0.014*
**Place of residence**				
Urban	49.1	31.5	26.7	17.7
Rural	50.9	45.5	28.4	19.3
P		0.009*	0.045*	0.254
**Community-level illiteracy**				
Low	59.6	31.3	28.2	26.6
High	40.4	39.2	26.3	18.4
P		0.001*	0.033*	<0.001*
**Community-level media use**				
Low	41.1	36.7	27.1	18.8
High	58.9	31.4	27.9	22.8
P		0.027*	0.336	0.003*
**Community-level poverty**				
Low	62.1	31.6	27.2	27.8
High	37.9	25.7	29.3	18.2
P		0.115	0.033*	<0.001*
**Geopolitical zones**				
North West	24.2	34.5	28.6	13.1
North East	15.4	30.5	31.6	28.8
North Central	19.5	30.4	28.3	21.7
South East	13.2	17.2	20.2	12.0
South South	14.7	22.6	26.1	3.3
South West	13.0	46.7	43.2	29.6
P		<0.001*	<0.001*	<0.001*

P-value obtained from Chi-square test; * Significant at p < 0.05.

**Fig 1:**
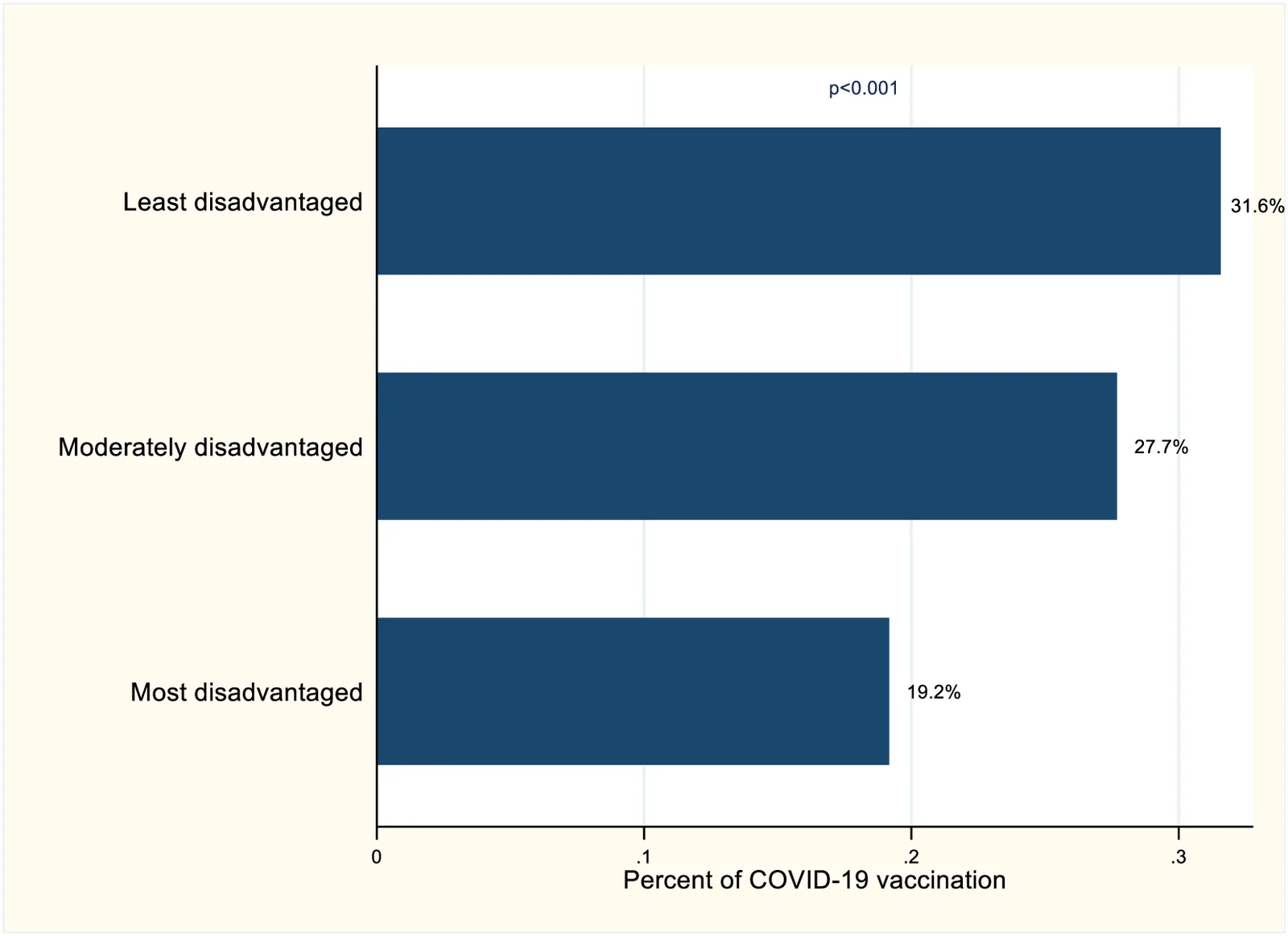
Distribution of COVID-19 vaccination by socioeconomic gradient.

Fig 1 shows women’s COVID-19 vaccination uptake by socioeconomic status. The least disadvantaged women had the highest COVID-19 vaccination uptake (31.6%), while the most disadvantaged women had the lowest COVID-19 vaccination uptake (19.2%). The proportions of COVID-19 vaccination uptake across socioeconomic status were statistically significant (p < 0.001).

**Fig 2:**
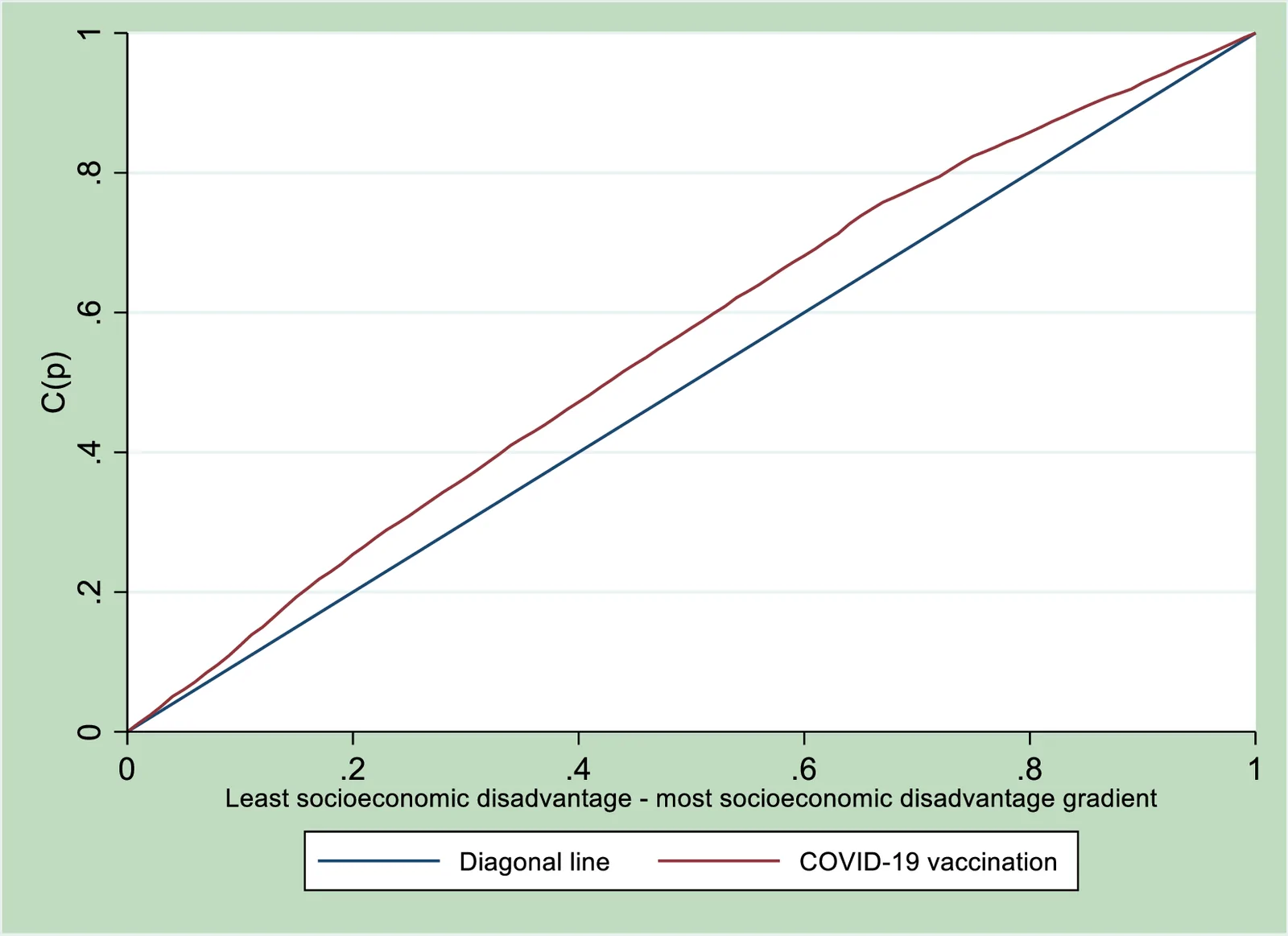
Concentration curve for COVID-19 vaccination by socioeconomic gradient.

Fig 2 shows the socioeconomic inequality in COVID-19 vaccination uptake among Nigerian women. How far the curve deviated from the diagonal line (line of equality) indicated whether there was inequality in COVID-19 vaccination uptake and to what extent. The figure shows that the least disadvantaged women had higher COVID-19 vaccination uptake as the curve sagged above the diagonal line (line of equality).

Table 2 shows the results of socioeconomic inequality in COVID-19 vaccination uptake. Overall, COVID-19 vaccination uptake was significantly more concentrated among the least disadvantaged women (Conc. Index = −0.110; SE = 0.005; p < 0.001). The analysis also revealed that significant concentration indices were observed in COVID-19 vaccination uptake across the levels of all factors, indicating socioeconomic inequality in COVID-19 vaccination uptake leaning towards the least disadvantaged women. The inequalities observed across each levels of women’s characteristics were statistically significant (p < 0.001) respectively, except in wife beating justified factor (p = 0.113).

**Table 2:** Concentration indices for COVID-19 vaccination uptake among Nigerian women.

Variable	Concentration Index (SE)	P
**Age in 5-year groups**		<0.001*
15-19	−0.042 (0.014)*	
20-24	−0.124 (0.012)*	
25-29	−0.110 (0.012)*	
30-34	−0.081 (0.012)*	
35-39	−0.105 (0.012)*	
40-44	−0.115 (0.012)*	
45-49	−0.113 (0.014)*	
**Highest educational level**		<0.001*
No education	−0.071 (0.008)*	
Primary	−0.063 (0.007)*	
Secondary	−0.016 (0.007)*	
Higher	−0.003 (0.008)	
**Religion**		<0.001*
Christianity	−0.018 (0.006)*	
Islam	−0.175 (0.007)*	
Traditiona/no religion	−0.150 (0.089)	
**Covered by health insurance**		<0.001*
No	−0.101 (0.005)*	
Yes	−0.005 (0.015)	
**Marital status**		<0.001*
Single	−0.060 (0.010)*	
Currently married	−0.138 (0.006)*	
Formerly married	−0.044 (0.018)*	
**Employment status**		0.031*
Not working	−0.092 (0.009)*	
Working	−0.070 (0.005)*	
**Wife beating justified**		0.113
No	−0.095 (0.005)*	
Yes	−0.114 (0.011)*	
**Exposed to media**		<0.001*
No	−0.099 (0.008)*	
Yes	−0.041 (0.005)*	
**Sex of household head**		<0.001*
Male	−0.112 (0.005)*	
Female	−0.061 (0.009)*	
**Household wealth**		<0.001*
Poorest	−0.053 (0.008)*	
Poorer	−0.077 (0.012)*	
Middle	−0.028 (0.010)*	
Richer	−0.057 (0.008)*	
Richest	−0.021 (0.008)*	
**Household size**		0.016*
1-2	−0.123 (0.014)*	
3-4	−0.113 (0.009)*	
5-6	−0.087 (0.009)*	
7+	−0.083 (0.008)*	
**Community-level acceptance of wife-beating**		<0.001*
More acceptance (less empowered)	−0.115 (0.008)*	
Less acceptance (more empowered)	−0.061 (0.006)*	
**Community-level willingness for COVID-19 vaccine uptake**		<0.001*
Low	−0.161 (0.010)*	
High	−0.086 (0.005)*	
**Community-level nativity**		<0.001*
Low	−0.057 (0.008)*	
High	−0.141 (0.006)*	
**Place of residence**		<0.001*
Urban	−0.049 (0.005)*	
Rural	−0.098 (0.007)*	
**Community-level illiteracy**		<0.001*
Low	−0.028 (0.005)*	
High	−0.090 (0.007)*	
**Community-level media use**		<0.001*
Low	−0.085 (0.007)*	
High	−0.037 (0.005)*	
**Community-level poverty**		<0.001*
Low	−0.036 (0.005)*	
High	−0.088 (0.007)*	
**Geopolitical zone**		<0.001*
North West	−0.209 (0.010)*	
North East	−0.020 (0.010)*	
North Central	−0.068 (0.011)*	
South East	0.013 (0.016)	
South South	0.023 (0.012)*	
South West	−0.018 (0.006)*	
**Total estimate**	−0.110 (0.005)*	

* Significant at p < 0.05; P = comparing concentration indices across the levels of a variable.

Table 3 shows the decomposition analysis of socioeconomic inequality in COVID-19 vaccination uptake among Nigerian women. The percentage contribution denotes the proportion of overall inequality that each factor accounts for. If a factor has a positive percentage contribution, it implies increasing the observed inequality, while the negative percentage contribution decreases the observed inequality. Having higher education (32.1869%), secondary education (30.6881%), exposed to mass media (17.7172%), employed (13.5692%), covered by health insurance (3.9903%), being aged 30 years or older, from South West (33.2156%), South South (9.2048%) and South East (4.2669%) respectively, were positive contributors to socioeconomic inequality in COVID-19 vaccination uptake. On the other hand, being from the North West (−19.3709%), rural residence (−12.9252%) and Christian faith (−8.9064%) made negative contributions to the overall socioeconomic inequality in COVID-19 vaccination uptake. This indicates that the distribution of these characteristics across socioeconomic groups, in combination with their associations with vaccination uptake, acted to offset the overall measured socioeconomic inequality. Thus, rather than increasing the observed inequality, these factors counterbalanced a portion of the inequality attributable to other determinants.

**Table 3:** Decomposition results of COVID-19 vaccination uptake by women’s characteristics.

Variable	Elasticity	Concentration Index	Absolute Contribution	% Contribution
**Age in 5-year groups**				
15-19	Reference			
20-24	0.0104	0.0437	0.0018	−1.6467
25-29	0.0116	0.0414	0.0019	−1.7495
30-34	0.0156	0.0105	0.0007	−0.5947
35-39	0.0150	−0.0448	−0.0027	2.4428
40-44	0.0144	−0.0698	−0.0040	3.6626
45-49	0.0123	−0.0663	−0.0033	2.9522
**Highest educational level**				
No education	Reference			
Primary	0.0061	0.0330	0.0008	−0.7324
Secondary	0.0383	−0.2210	−0.0338	30.6881
Higher	0.0228	−0.3882	−0.0355	32.1869
**Religion**				
Christianity	Reference			
Islam	0.0091	0.2704	0.0098	−8.9064
Traditiona/no religion	−0.0003	0.2059	−0.0003	0.2531
**Covered by health insurance**				
No	Reference			
Yes	0.0029	−0.3787	−0.0044	3.9903
**Marital status**				
Single	Reference			
Currently married	0.0017	0.0795	0.0005	−0.4824
Formerly married	0.0005	−0.1381	−0.0003	0.2590
**Employment status**				
Not working	Reference			
Working	0.0338	−0.1107	−0.0150	13.5692
**Wife beating justified**				
No	Reference			
Yes	0.0008	0.2600	0.0009	−0.7993
**Exposed to media**				
No	Reference			
Yes	0.0224	−0.2181	−0.0195	17.7172
**Sex of household head**				
Male	Reference			
Female	0.0019	−0.2506	−0.0020	1.7712
**Household wealth**				
Poorest	Reference			
Poorer	0.0030	0.3993	0.0049	−4.4040
Middle	0.0075	0.0287	0.0009	−0.7832
Richer	0.0088	−0.2872	−0.0101	9.1928
Richest	0.0013	−0.4604	−0.0023	2.0976
**Household size**				
1-2	Reference			
3-4	−0.0011	−0.01147	0.0005	−0.4471
5-6	−0.0005	−0.0669	0.0001	−0.1245
7+	−0.0007	0.1782	−0.0005	0.4467
**Community-level acceptance of wife-beating**				
More acceptance (less empowered)	Reference			
Less acceptance (more empowered)	0.0127	−0.2160	−0.0110	9.9378
**Community-level willingness for COVID-19 vaccine uptake**				
Low	Reference			
High	0.1105	0.0008	0.0004	−0.3342
**Community-level nativity**				
Low	Reference			
High	0.0160	0.01374	0.0088	−7.9895
**Place of residence**				
Urban	Reference			
Rural	0.0088	0.4031	0.0142	−12.9252
**Community-level illiteracy**				
Low	Reference			
High	0.0040	0.4775	0.0077	−6.9749
**Community-level media use**				
Low	Reference			
High	−0.0015	−0.3344	0.0020	−1.8449
**Community-level poverty**				
Low	Reference			
High	0.0018	0.5204	0.0037	−3.3366
**Geopolitical zone**				
North West	Reference			
North East	0.0148	0.3597	0.0213	−19.3709
North Central	0.0118	0.0593	0.0028	−2.5459
South East	0.0051	−0.2327	−0.0047	4.2669
South South	0.0061	−0.4180	−0.0101	9.2048
South West	0.0172	−0.5328	−0.0366	33.2156

## Discussion

There were differences in the uptake of COVID-19 vaccination among reproductive-age Nigerian women. Approximately one-third of the least disadvantaged, one-quarter of the moderately disadvantaged, and one-fifth of the most disadvantaged, respectively reported receiving COVID-19 vaccination. This is less than the estimate of uptake that have been reported among women in other parts of the world [41,42]. The observed low uptake of COVID-19 vaccination among Nigerian women could be due to persistent gaps in access to health interventions including vaccination as commonly reported in Nigeria [5,6,43]. The disparity in uptake across socioeconomic strata, with higher uptake among the least disadvantaged women, compared to the most disadvantaged counterparts, may be associated with differences in vaccination coverage across socioeconomic gradient [5]. This pattern is consistent with previous evidence that wealthier individuals have correlation with higher uptake or utilization of health services [19,44,45]. Whereas, the most disadvantaged women could experience barrier in access and use due to limited resources for transportation to health facility, cost for services, and poor health literacy.

Moreover, the wealthy individuals are more likely to reside in urban areas with better healthcare infrastructure, greater exposure to health information, and possess higher autonomy in health-related decision-making. These factors may be associated with access to and acceptance of vaccination. The findings from this study showed inequitable COVID-19 vaccination uptake between the least disadvantaged and most disadvantaged women, suggesting that current vaccination programme may not have adequately reached the most disadvantaged populations. To address this disparity requires targeted, equity-focused programme approaches, including community-based outreach, improved health literacy campaign, and the provision of financial and transportation resources to ensure higher COVID-19 vaccination coverage.

The negative and statistically significant concentration index (CI = −0.110; SE = 0.005; p < 0.001) provide evidence that the least disadvantaged women have higher COVID-19 vaccination uptake, indicating a disproportionate coverage pattern. The magnitude of the index suggests a meaningful degree of inequality, while the small standard error and strong statistical significance indicate precision of the estimate. This pattern implies that socioeconomic status may be associated with access to and utilization of COVID-19 vaccination [46–49]. The significant concentration indices across all examined covariates further underscores the systemic nature of this inequality. It suggests that disparities are not confined to a single dimension, but rather cut across multiple intersecting factors such as education, residence, and access to healthcare [48,49]. These findings may reflect the structural inequities within the health system, where the well-off may be better positioned to benefit from public health interventions.

The decomposition results provide important insights into the drivers of socioeconomic inequality in COVID-19 vaccination uptake among Nigerian women. The factors with positive percentage contributions, such as higher (32.19%) and secondary education (30.69%), exposure to mass media (17.72%), employment status (13.57%), and health insurance coverage (3.99%), substantially increased inequality in COVID-19 vaccination uptake across disadvantaged groups. These factors are should be considered when designing vaccination programmes that are focused to achieve universal coverage. Similarly, zonal contributions from the South West (33.22%), South South (9.20%), and South East (4.27%) suggest that geographic disparities in health infrastructure and service delivery further widened the gap in inequality. Overall, these findings show the correlation that inequality may be driven by both compositional (distribution of characteristics) and structural factors, emphasizing the need for targeted, context-specific interventions [46–49].

### Strengths and limitations

The robust, nationally representative dataset used in this study was gathered by the 2024 NDHS. The decomposition analytical technique employed in this study, accounts for the socioeconomic inequalities in COVID-19 vaccination uptake among reproductive-age Nigerian women. Nonetheless, the use of self-reported COVID-19 vaccination status may have introduced recall, misclassification, residual confounding, and social desirability bias, potentially affecting the accuracy of the study findings. Recall bias could have resulted in some respondents misremembering whether they had received a vaccine, particularly if vaccination occurred several months before the survey or if participants received multiple doses. Social desirability bias may have led some women to report vaccination to conform to perceived expectations, especially in settings where vaccination was promoted by government and health authorities. Importantly, the implications for estimated socioeconomic inequalities depend on whether misclassification was differential across socioeconomic groups. If reporting errors were broadly similar across wealth, education, or residence categories, the estimates of inequality may have been attenuated but remain directionally valid. However, if more advantaged women were more likely to accurately recall or socially report vaccination than disadvantaged women, the observed inequalities could have been exaggerated. Conversely, differential under-reporting among advantaged groups could have underestimated inequality. Thus, self-reporting may have affected the magnitude and, potentially, the validity of inequality estimates. The differential misclassification by socioeconomic status could bias the inequality estimates in either direction, and this could limit the precision of COVID-19 vaccination estimates. In addition, due to the cross-sectional nature of the study design, only association can be established and not causal inference. There is possible measurement error from constructed community-level variables.

## Conclusion

This study identified differences in COVID-19 vaccination uptake across socioeconomic groups. The least disadvantaged women had the highest COVID-19 vaccination uptake. While COVID-19 vaccines offer a critical pathway to controlling the pandemic, its benefits cannot be fully realized without equitable uptake across all population groups. In Nigeria, persistent socioeconomic inequalities pose significant challenges to achieving this goal. Women, particularly those from disadvantaged backgrounds, may be facing greater barriers that limit their access to vaccination services. Therefore, this is a timely and necessary research focusing on socioeconomic inequality in COVID-19 vaccination uptake among Nigerian women. This will not only enhance understanding of the drivers of inequity but also support the development of targeted strategies to improve vaccination coverage and promote health equity in Nigeria.

## Supporting information

S1 Checklist STROBE checklist cross-sectional. (DOC)
